# Mild Mechanic Stimulate on Acupoints Regulation of CGRP-Positive Cells and Microglia Morphology in Spinal Cord of Sciatic Nerve Injured Rats

**DOI:** 10.3389/fnint.2019.00058

**Published:** 2019-11-14

**Authors:** Li Yi-zhen, Miao Run-pei, Yu Tian-yuan, Bai Wan-zhu, Cui Jing-jing, Lu Meng-qian, Shen Yi, Luo Yu-ting, Shao Shuai, Zhang Yu-mo, Mo Yan-jun, Lv Tao-tao, Chen Guo-yong

**Affiliations:** ^1^School of Acupuncture-Moxibustion and Tuina, Beijing University of Chinese Medicine, Beijing, China; ^2^Dongzhimen Hospital, Beijing University of Chinese Medicine, Beijing, China; ^3^Institute of Acupuncture and Moxibustion, China Academy of Chinese Medical Sciences, Beijing, China; ^4^Yidu Cloud Technology Inc., Beijing, China

**Keywords:** sciatic nerve injury, nerve beam tracer, microglia, CGRP, nerve regeneration and repair, acupuncture

## Abstract

**Objective:**

The objective of this study is to observe the effects of mild mechanical stimulation on acupuncture points of spinal motor neurons and active substances of sciatic nerve injury in rats, and to explore the morphological basis for the recovery of motor function in rats with sciatic nerve injury, using mild acupuncture. Acupuncture in the local area of injury may cause further damage to the peripheral nerve injury. We believe that mild mechanical stimulation on the surface, using some specific acupuncture points can also have a positive effect on nerve repair. This method, called Chinese tuina, has existed for more than 2,000 years in China.

**Methods:**

This study establishes a rat model using sciatic nerve crush injury. Rats received Chinese tuina in accordance with the principle of the three methods and three points, once a day, for 20 days. The rats’ status of hindlimb recovery was detected by a sciatic functional index. The labeled neuronal cell body was used to evaluate the fiber recovery after the rats’ sciatic nerve injury, using a neural tracing technique. Our team studied motor neuronal cell bodies, CGRP-positive cells, and the microglia of damaged sciatic nerves which were stained with fluorescent triple staining, adopting a confocal multi-layer scanning technique, and then the changes in neuronal activity distribution and expression, and changes of time and treatment were described, using the method of morphological description.

**Results:**

Sciatic nerve injury decreased the survival rate of motor neurons, affected CGRP-positive cells, and activated microglia in the ventral horn of the spinal cord. Compared with the model group, the survival of spinal ventral horn motor neurons was increased through tuina intervention. The swelling of CGRP-positive cells was alleviated, and the degree of microglia activation was less than that of the model group.

**Conclusion:**

This study used visual morphological findings to assess changes in neurons and active substances with time after injury of the peripheral nerve, and demonstrated that peripheral mild acupuncture intervention improved the capacity of neurofibrillary axoplasmic transport, regulated microglia activation, and significantly promoted the recovery of sciatic nerve injury.

## Introduction

Peripheral nerve injury (PNI) is a common cause of illness among disabled and young people. PNIs are becoming increasingly familiar due to the increasing frequency of trauma incidents resulting from motor vehicle accidents. Fracture injuries and crush injuries are common in clinical practice, mostly due to earthquakes, tsunamis, traffic accidents, and surgery. In patients with multiple traumas, the rate of peripheral nerve injury is estimated to be 2.8%, with brachial plexus involvement reaching 5% (Rakolta and Omer, 1969; Omer, 1974; Noble et al., 1998). PNI may cause pain, numbness, and other motor dysfunction in muscles and tissues innervated by damaged nerves. Injuries caused by surgical procedures is also an important source of PNI (Tasuku et al., 2015; Jang et al., 2016). At the same time, peripheral nerve injury often occurs in chronic diseases such as cervical spondylosis and lumbar disk herniation, which causes sensory and motor dysfunction through the compression of nerves such as in the bone or the intervertebral disk.

Damage to neuronal axons and cell bodies is an important part of nerve repair after peripheral nerve injury. There are mainly two types of motor neurons in the ventral horn of the spinal cord called α and γ, which control voluntary movement. By observing the changes in the number and morphology of the neurons, we can know the completeness and neurological status of nerve fibers. The Calcitonin gene-related peptide (CGRP) plays an important role in the transmission and regulation of pain information (Yu et al., 2009; Shi et al., 2012). As an important item of biochemistry, it is used to show the changes in pain and is also used to evaluate the role of acupuncture on the modulation of the pain syndrome. The activity of CGRP can determine the neurological status in the ventral horn of the spinal cord after sciatic nerve crush injury (Li et al., 2004; Shi et al., 2010).

The original name of Chinese tuina, a mild mechanical stimulation on acupuncture points, at the time of its inception 2000 years ago, was anqia massage. It is regarded as a symbol of both naturopathy and physical therapy (Xu, 2013) and one of the earliest Chinese clinical treatments. Guided by the theory of Chinese medicine, Chinese tuina is performed *via* either hand manipulations or massage implementations on certain parts or points of the patient’s body (Yu, 2015). This treatment method is well established and has a wide range of clinical applications (Li et al., 2012). In China, tuina has long been used and will continue to be used as a common method to treat sensory and motor dysfunction and diseases caused by peripheral nerve injury, including cervical spondylosis (Wen et al., 2015; Hu et al., 2016) and the prolapse of the lumbar intervertebral disk (Chen et al., 2016). Three methods and three points means that point pressing, strumming, and kneading manipulation, the most common sub methods of tuina, works on the three most common acupuncture points—*Yinmen* (BL37), *Chengshan* (BL57), and *Yang-lingquan* (GB34)—to treat peripheral nerve injury (Guo et al., 2016). Findings of our group support that these methods can significantly improve the motor-related functional manifestations of SNI rats, including the recovery of fine movements tested by the sciatic nerve function index and the recovery of hind limb muscle strength, tested using the swash plate test. Prior to intervention, a significant decrease in the swash plate test was detected in the model group compared with the normal group. On day 20 post-intervention, swash plate tests of the tuina group were significantly increased compared with the model group, while being similar to the normal group (Li et al., 2018). Furthermore, tuina manipulation promoted the increase of nerve growth factor, p75 neurotrophin receptor, TrkA (Mei et al., 2013), MAP-2, NT-3, and NF-M (Gao et al., 2014) in the spinal cord.

This study focuses on the morphology of the spinal cord and elucidates the mechanism of Chinese tuina during the treatment of a sciatic nerve crush injury. A neuronal tract tracing technique was used to observe the integrity of spinal motor neurons. At the same time, to visualize the morphological changes of nerve cells in the spinal cord by tuina interventions, Immunofluorescence staining was used to observe CGRP which are closely related to neurons and the microglia marker CD11b.

## Materials and Methods

### Group Assignment

The protocols were conducted in compliance with the Guidance Suggestions for the Care and Use of Laboratory Animals, formulated by the National Institute of Health. All experimental procedures were approved by the Medical and Experimental Animal Ethics Committee at Beijing University of Chinese Medicine (BUCM-4-2018101902-4010). Sixty-four male specific pathogen-free Sprague-Dawley rats (Adamas Beifu, Beijing, China; SCXK (Jing) 2016-0002) aged 6–7 weeks and weighing 200 ± 10 g were raised at 23 ± 2°C and 45% humidity, with a 12 h light/dark cycle (lights were turned on at 8:00 a.m.) and were allowed free access to food and water. All interventions on the various groups were performed between 8:00 a.m. and 12:00 a.m. The number of rats used, and their discomfort were minimized as much as possible.

The rats were randomly put into four groups: 16 rats for the normal group, 48 rats for sciatic nerve crush injury intervention group, including: 16 model rats requiring no intervention, 16 model rats as the control group who were bound controlled (Tying the small board to the right lower limb of the rat with a rope) once a day (9 min/day) for 20 days, starting from the 7th day after the surgery; and 16 rats in the tuina group that were given tuina therapy once a day (9 min/day) for 20 days, starting from the 7th day after the surgery.

### Establishment of Sciatic Nerve Injury Models

**FIGURE 1:**
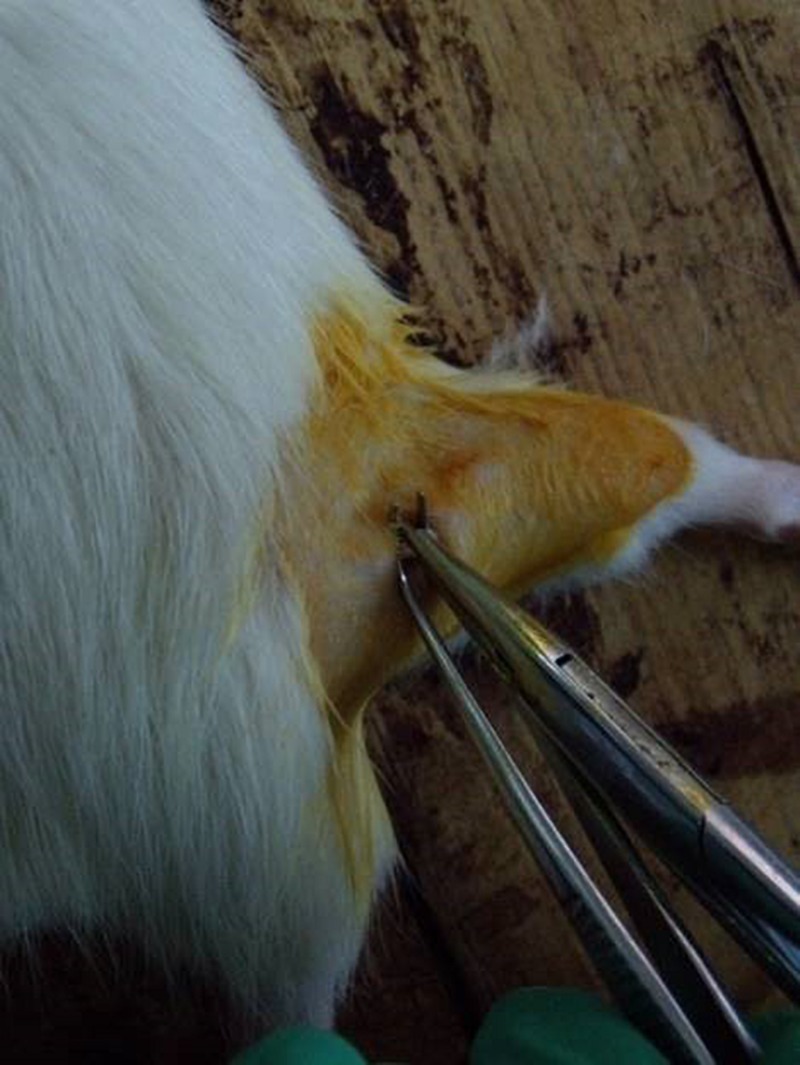
Sciatic nerve injury model operation.

The rats underwent fasting and water deprivation 24 h before the surgery. They were intraperitoneally anesthetized with a premixed solution which had 10% chloral hydrate (350 mg/kg body weight). The right lateral thigh of the rats were shaved and the uncovered skin sterilized with 10% povidone iodine. A gluteal muscle-splitting incision exposed the right sciatic nerve (Liu H. et al., 2010). Based on Sunderland’s classification grade III nerve injury (Sunderland, 1981) of peripheral nerve injury and calculations of pressure intensity, a pair of non-serrated forceps exposed the crush sciatic nerves at the mid-thigh level for 30 s (Figure 1). The skin was then sutured with four stitches. After the operation, the rats were given no food but water for 24 h.

### Walking Track Analysis

Rats were tested in a confined walkway measuring 80 cm long and 10 cm wide, with a dark shelter at the end. A white paper was placed on the floor of the rat walking corridor. The hind paws of the rats were pressed down onto a finger paint-soaked sponge, and the rats were then allowed to walk down the corridor leaving their hind footprints on the paper. Several measurements were taken from the footprints: (1) distance from the base of the heel to the tip of the third toe, the print length (PL); (2) distance from the first to the fifth toe, the toe spread (TS); and (3) the distance from the second to the fourth toe, the intermediary toe spread (IT). All three measurements were taken on the experimental (E) and normal (N) sides. The sciatic functional index (SFI) was calculated as described by Bain et al. (1989) according to the following equation:

$$\begin{array}{lll} SFI & = & -38.3[(EPL-NPL)/NPL]\\ & & +109.5[(ETS-NTS)/NTS]\\ & & +13.3[(EIT-NIT)/NIT]-8.8 \end{array}$$

The SFI oscillates around 0 for normal nerve function, and −100 or less represents total dysfunction. Records of the footprints were obtained before surgery and on the following days post-treatment: day 0, 5, 10, 15 and 20.

### Microinjection of FG

**FIGURE 2:**
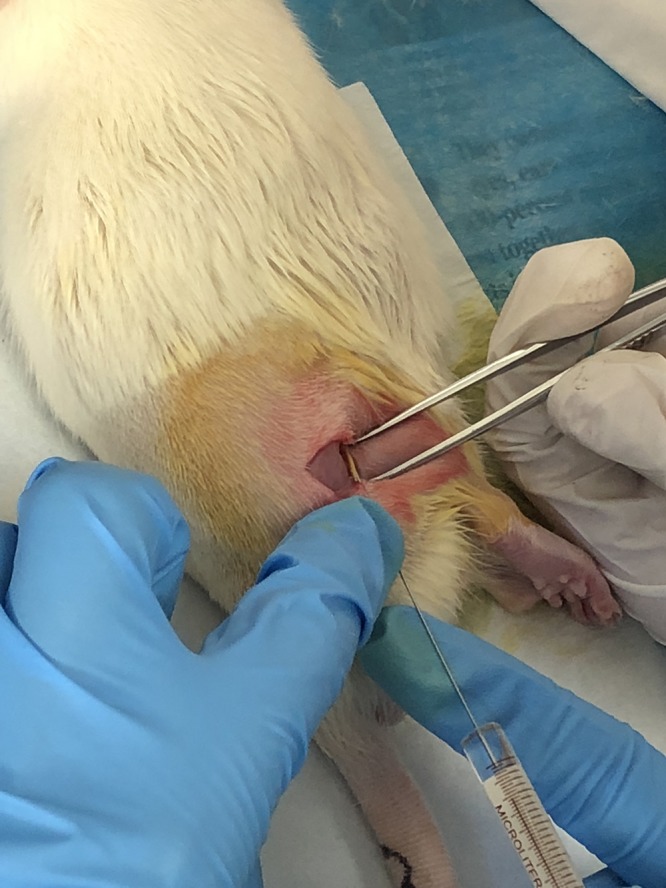
Sciatic nerve tracer injection.

The Fluoro-Gold tracer injection surgery was carried out 3 days before the rats were sacrificed (Figure 2). Fasting and water deprivation were conducted for 12 h prior to surgery. The right lateral thigh was shaved, and the skin was disinfected with 10% povidone iodine. Under anesthesia with ether, the right sciatic nerve was exposed using the gluteal-splitting approach (Liu H. et al., 2010). FG (Fluoro-Gold) was used as a retrograde tracer to determine the distribution of neurons related to the sciatic nerve. FG (0.5 μl) was slowly injected into the point below the sciatic nerve injury for 1 cm and rapid local yellowing occurred. In order to prevent leakage of the solution, the needle remained inserted 1 min after injection and was then slowly pulled out, and the injection point was gently rubbed with a sterile cotton swab. Subsequently, the skin was sutured with four stitches. The awake rats were then put back into the raising box.

### Tuina Treatment

**FIGURE 3:**
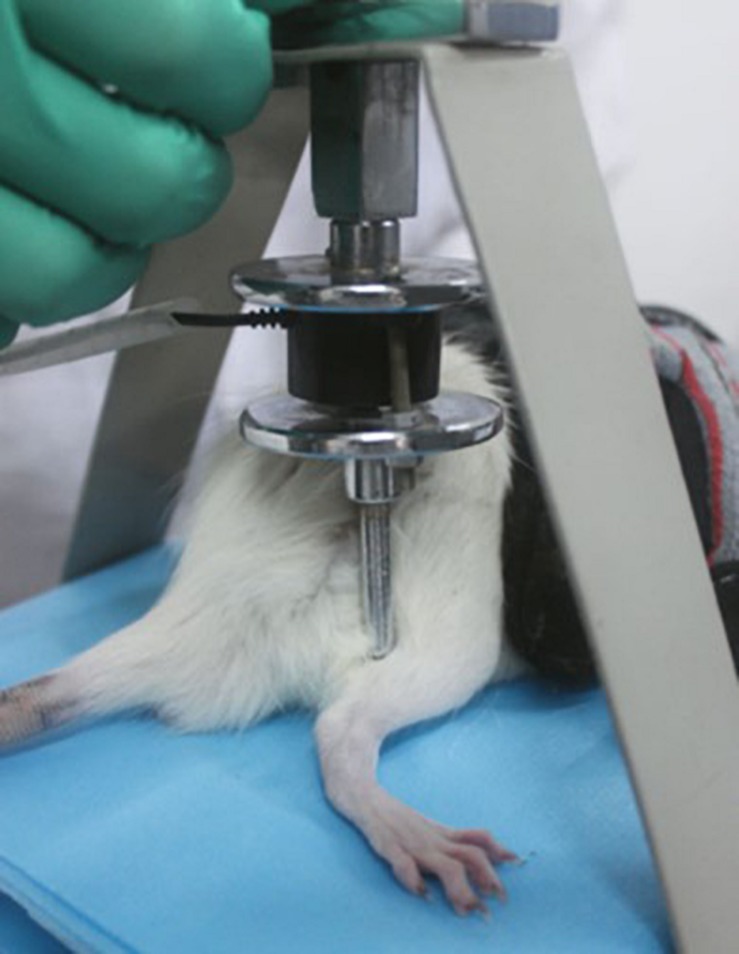
Tuina manipulation emulator.

In 2007, a tuina manipulation emulator (patent No. ZL200710187403.1) (Figure 3) was designed by our team, hoping to stimulate tuina techniques and simultaneously to maintain qualitative and quantitative control. The essential parts of the emulator comprises a contact point, disk, and stepper motor. A metallic strip connects the pressure sensor and the contact point, which guaranteed sensor’s sensitivity and the ideal condition so that no abrasion is induced by long-term use. A lead screw is used to adjust the amount of pressure applied at the contact point (a 10 mm cylinder), which is displayed on the control screen. According to the *Three Methods and Three Points*, the emulator is used, with a frequency of 30 times/min and 0.98 N of a force, to perform the point pressing, strumming, and kneading manipulation on *Yinmen* (BL37), *Chengshan* (BL57), and *Yang-lingquan* (GB34) sequentially on the affected site, in other words, using three different tuina methods at three acupuncture points. Each tuina method lasted for 1 min on each acupuncture point, respectively. Seven days after the sciatic nerve injury models were established, these methods were performed once a day for 20 days. Based on the principle of comparative anatomy, *Yinmen* (BL37) is pinpointed on the back of the thigh, 3/7 down the line connecting the midpoint of the buttocks fold and the center of the popliteal fossa, *Chengshan* (BL57) in the center of the posterior of the leg, between the two heads of the gastrocnemius, and *Yang-lingquan* (GB34) in the depression anterior and inferior to the fibular capitulum.

### Morphological Observation

At 7 and 27 days after sciatic nerve injury, morphology on CGRP and CD11b of the spinal cord was observed in rats from each group. After anesthesia with chloral hydrate and intracardial perfusion with normal saline and 4% paraformaldehyde, the spinal cords were immersed in the 4% paraformaldehyde for 2 h and then placed into 25% sucrose in 0.1 mol/L PB solution for 48 h. Transverse sections (40 μm) were cut using a rotary microtome (Thermo, Microm International GmbH, Germany). The membranes were blocked in 3% donkey serum for 1 h, and then incubated with primary antibodies against CGRP (1:1000; No. ab81887, Abcam, United Kingdom), CD11b (1:1000; No. MCA275GA, Bio-Rad, United States) and FG (1:1000; AB153-1; Chemicon, Temecula, CA,EUA) at 4°C overnight. Afterward, the sections were washed with 0.1 mol/L PBS three times, exposed to goat anti-mouse Alexa 488 and Alexa 594 secondary antibody (1:500; Invitrogen-Molecular Probes, Eugene ORUSA), Neuronal marker Nissl (1:1000; NeuroTrace 435/455, United States) at room temperature for 1 h. After that, the sections were mounted on microscope slides and plated with glycerol (glycerol: pure water = 1:1), the tissue was then placed in a cassette and refrigerated at 4°C for use. Different fluorescence is generated under a fluorescence microscope (Y-IDP; Nikon Co., Tokyo, Japan) according to different substances, and the color filter system is replaced by different excitation wavelengths of different fluorescein, while the wavelength of the emitted light is different. Photographs showed the fluorescence excited by different wavelengths through the camera system (DMX1200C; Nikon, Japan). Digital images were finally processed with Adobe Photoshop CS2 (Adobe Systems, San Jose, CA, United States). Under the corresponding exciting light in the fluorescence microscope system, FG labeled neurons appeared green, Alexa 594 labeled CD11b, CGRP-positive neurons and axonal terminals in red, and labeled nissl body in blue.

### Statistical Analysis

Data, expressed as means ± standard error of mean (SEM), were analyzed using SPSS 20.0 software (SPSS Inc., Chicago, IL, United States). When the data variances were equal, one-way ANOVA data analysis of variance and *post hoc* least significant difference test was used to analyze data that obeyed normal distribution and homogeneity of variance. When the data variances were not equal, we used non-parametric test to analyze data. A *P*-value less than 0.05 was considered to be statistically significant.

## Results

### Effects of Tuina Treatment on SFI

**FIGURE 4:**
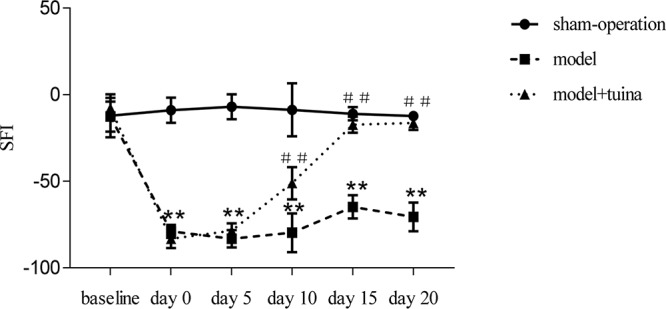
Assessment of SFI in Rats Subjected to Sciatic Nerve Crush Injury ($\bar{\text{X}}$ ± SEM, *n* = 8). ^∗∗^*P* < 0.01, compared with the sham-operated group, ^##^*P* < 0.01, compared with the model group.

The baseline SFI values were similar for all three groups of rats prior to surgery (Figure 4). However, a remarkable reduction (*P* < 0.01) in SFI was observed in animals subjected to sciatic nerve crush injury in the model and model + tuina groups compared with the sham-operated group −78.91 ± 3.73 and −83.13 ± 5.38 vs. −8.91 ± 7.26, respectively) 1 week post-operation (on treatment day 0). On the 5th day post-treatment, the model and model + tuina groups presented a recovering tendency, but remained significantly different (*F* = 461.90, *P* < 0.01) as compared with the sham-operated group (−83.15 ± 4.99 and −78.62 ± 4.25 vs. −6.99 ± 7.20, respectively). On the 10th day, the model + tuina group (−51.08 ± 9.33) showed a more obvious positive tendency than the model group (−79.74 ± 11.21). Compared with the sham-operated group (−8.77 ± 15.28), the differences were still statistically significant (*F* = 68.60, *P* < 0.01). On the 15th day post-treatment, there were significant differences (*F* = 258.72, *P* < 0.01) among the sham-operated (−11.03 ± 3.81), model (−64.80 ± 6.68) and model + tuina groups (−17.32 ± 4.59). The model + tuina group showed significant improvement compared with the model group (*P* < 0.01). After 20 days of treatment, the SFI of the model group was still significantly lower (*F* = 288.91, *P* < 0.01) compared with that of the sham-operated group (−70.56 ± 8.25 vs. −12.33 ± 2.13, respectively), and the model + tuina group showed no significant differences compared with that of the sham-operated group, and significant differences were found between the model and model + tuina groups as well (*P* < 0.01).

### The CGRP Expression on the Motor Neurons

**FIGURE 5:**
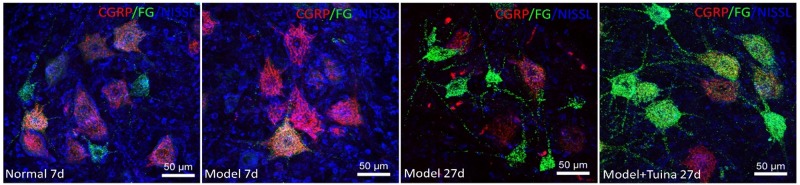
The CGRP expression on the motor neurons. Notes: a representative transverse section from the lumbar ventral horn of the spinal cord showing the labeled cells of CGRP, the labeled cells of CGRP prior to intervention and on day 20 post-intervention (Magnification is 400^∗^).

As shown in Figure 5, prior to intervention, in the normal group, CGRP were mainly distributed in ventral horn motor neurons. The ventral horn cells are mostly large cells, the CGRP-positive substances are mainly located in the cytoplasm and the dendrites are short. In the model group, the CGRP-positive substances are deeply deposited in the protrusions, and the cell outline is clear. On day 20 post-intervention, CGRP-positive cell expression decreased in the tuina group compared with the model group. At the same time, with the prolongation of injury time and the swelling of nissl-labeled motor neurons increased, and the positive CGRP staining in cytoplasm also became deeper. Compared with the model group, the swelling of neurons in the tuina group was reduced, and the activity of CGRP-positive cells was decreased.

### Microglial Activation Around the Motor Neurons

**FIGURE 6:**
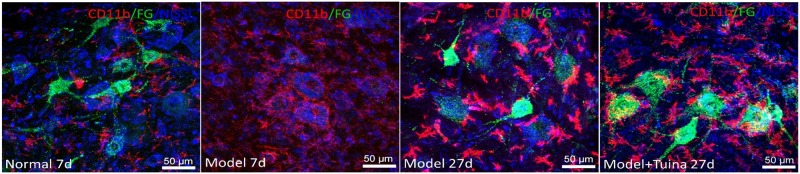
Microglial activation around the motor neurons. Notes: a representative transverse section from the lumbar ventral horn of the spinal cord showing the labeled cells of microglia. The labeled cells of microglia prior to intervention and on day 20 post-intervention (Magnification is 400^∗^).

As shown in Figure 6, prior to intervention, compared with the normal group, CD11b-labeled microglia in the model group showed a distinct activation state, which characterized deepening of microglia coloration, and the increase of density on the injury side surrounding the damaged motor neurons. On day 20 post-intervention, the degree of activation of microglia labeled by the sciatic nerve injury in the model and tuina group was enhanced, and the coloration was significantly more deepened than that of the normal group. The number of FGs co-expressed in the tuina group was more than that in the model group. As the number of labeled neurons increased, the activity of microglia decreased. The types of active microglia in the model group were mainly amoeba and round, which were different from the form of a highly branched rod in the tuina group. Compared with the tuina group, the size of the microglia pair was increased and the cytoplasm was more rich, and it was mostly located around the neurons which was in close contact with the damaged sciatic nerves.

### The Changes of the Number of Motor Neurons

**FIGURE 7:**
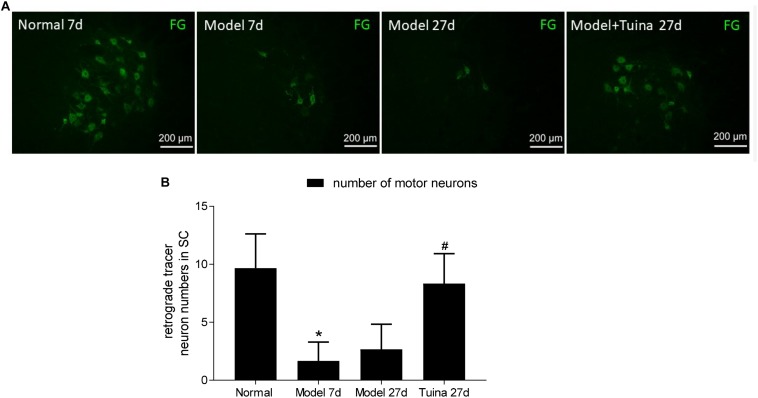
**(A)** Fluorescent gold-labeled motor neurons; **(B)** retrograde tracer neuron numbers in SC. The changes of the number of motor neurons. Data are expressed as the mean ± SEM (*n* = 6; one-way analysis of variance and *post hoc* least significant difference test). ^∗^*P* < 0.05, vs. normal group; #*P* < 0.05 vs. model group.

As shown in Figure 7, in the rats, the total of FG labeled motor neurons were counted in the ventral horn of the spinal cord (Figure 7A). All statistical data were obtained from L_4_-L_6_ spinal cord sections of each group, and six slices of one rat were selected from each group.

Prior to intervention, a significant decrease in the number of FG labeled motor neurons was detected in the ventral horn of the spinal cord with sciatic nerve crush injury (*P* < 0.05, vs. normal group). On day 20 post-intervention, the numbers in the tuina group was significantly increased compared with the model group (*P* < 0.05), whereas the numbers in the tuina group were similar to that in the normal group (Figure 7B).

## Discussion

Our previous studies showed that Chinese tuina significantly increased the expression of neurotrophic factors after sciatic nerve injury, obviously regulated the expression of CGRP in the spinal cord, and promoted the recovery of sensory function in rats (Xian et al., 2015). Chinese tuina also prevented depolymerization of neurofilaments and microtubules (Lu et al., 2015) and increased the expression of neurotrophic factors in the neural pathway (Mei et al., 2013). The mechanism of Chinese tuina for treating peripheral nerve injury has been initially elucidated.

Sciatic nerve injury is a common standard peripheral nerve injury model and is well-established as a method to investigate the impact of different treatments in neural injury repair (Ma et al., 2013). This method is relatively inexpensive, easy to perform, and its capacity for regeneration is equivalent for this type of injury in rats and subhuman primates (Marcolino et al., 2013).

In this study, the acupuncture points used for the intervention were *Yinmen* (BL37), *Chengshan* (BL57) and *Yanglingquan* (GB34). These acupuncture points are used because they are all related to neural tissue and muscle. In view of traditional Chinese acupuncture and moxibustion, which belong to parts of the bladder meridian of Foot-taiyang and the gallbladder meridian of Foot-shaoyang in the acupuncture system, meeting the principles of selecting acupuncture points with local and meridian areas to treat most diseases. From an anatomical point of view, *Yinmen* (BL37) is located in the sciatic nerve trunk, *Chengshan* (BL57) and *Yanglingquan* (GB34) are located on the branches of the sciatic nerve which are namely the phrenic nerve and the common peroneal nerve. There are three important pieces of muscle related to the sciatic nerve that are distributed around these three points, including the bicep muscle, gastrocnemius muscle, and tibialis anterior muscle, so these points are the best to stimulate the acupoint-nerve-muscle at the same time.

This present study mainly investigated the effects of tuina on the morphological changes of CGRP-positive cells and microglia in the ventral horn of the spinal cord. CGRP is an important neuron associated with pain formation and maintenance and is widely distributed in nervous tissues. Significant expression changes of CGRP after peripheral nerve injury may be involved in the early transmission of injury signals to the central nervous system and activate multiple pathways to induce nerve regeneration, which is a very important substance in nerve regeneration (Seybold et al., 2003). Experimental studies have shown that CGRP-positive cells are excitatory terminals that release excitatory neurotransmitters to enhance the action of downstream molecules. CGRP coexists with various neurotransmitters and neuropeptides, and is expressed in the peripheral and central nervous system (Ju et al., 1987; Tsujimoto and Kuno, 1988; Tan et al., 2008; Benarroch, 2011). For example, CGRP coexists with acetylcholine in motor nerve terminals for regulating classic neurotransmitters release (Tsujimoto and Kuno, 1988), and also coexists with various neurotransmitters in the sensory nervous system, such as substance P (Ju et al., 1987). Among some rats models established by chronic pain, CGRP-positive cells were densely distributed in the superficial region of the spinal cord and were significantly different from the normal group (Qiu et al., 2013). In DRG, CGRP-positive neurons participate in the sending sensory afferent input to nociceptive and viscerosensitive neurons in the spinal cord (Cui et al., 2015).

Microglia are normally in a resting state and are considered to be phagocytic cells in the central nervous system. When the central nervous system is damaged, microglia are activated, and plasticity changes in morphology and function (Streit et al., 2010). Microglia appear to proliferate and hypertrophy when the injury does not cause neuronal death, and the microglia is converted to phagocytic cells when neurotoxicity causes neuronal death (Kreutzberg, 1996). Round, amoebic, rod-shaped microglia represent fully activated microglia, and high-branched microglia represent partially activated microglia (Morioka et al., 1992). Activated microglia can release oxygen free radicals, nitric oxide, proteases, inflammatory cytokines and cause cytotoxic effects, and also secrete growth factors to support tissue repair (Liu B. et al., 2010). The degree of activation of microglia is one of the ways to determine the condition of neuronal injury. Sciatic nerve injury causes the corresponding ventral horn motor neurons and the nerve fibers of the spinal ganglia to send out a signal which activates microglia in the spinal cord. The activated microglia gathers around the injured neurons to produce neurotrophic factors which aim to promote the recovery of neurons. While the neuron is difficult to recover, another signal that triggers the production and release of microglial toxic substances will be sent out. The wrapped neurons are gradually phagocytosed by the glial cells to become colloidal scars to protect the surviving neurons.

This present study describes the morphological distribution and change trends of CGRP-positive cells and microglia in the ventral horn of the spinal cord. Tuina may promote sciatic nerve motor function recovery by regulating the activity of CGRP-positive cells and microglia in the ventral horn of the spinal cord. This finding provides a direct method to understanding the underlying regulating mechanisms of the neuropeptide and microglia, caused by a sciatic nerve injury. We provided morphological evidence for the treatment of peripheral nerve injury diseases using Chinese tuina. The statistical FG-labeled motor neurons were limited to the number of neurons positive in the same field of view.

The present study aims to supplement the evidence of the regulation of chemical active substances in the spinal cord using tuina treatment for sciatic nerve injured rats. Furthermore, on the basis of this study, we will analyze the secreted and activated products of microglia activation quantitatively, to observe whether there is a closer relationship between neuronal recovery and the products, and to further explore the mechanism of tuina in promoting the recovery of motor function in SNI rats.

## Data Availability Statement

The raw data supporting the conclusions of this manuscript will be made available by the authors, without undue reservation, to any qualified researcher.

## Ethics Statement

Medical and Experimental Animal Ethics Committee at Beijing University of Chinese Medicine.

## Author Contributions

LY-Z drafted the manuscript. YT-Y and BW-Z participated in the design of the study, and guided the study and manuscript. LY-T and CJ-J provided assistance for the laboratory research. LY-Z and MR-P translated the manuscript from Chinese, and performed the figure analysis and statistical analysis. SY carried out the figures. MY-J, ZY-M, SS, and LT-T provided assistance for animal research. LM-Q and CG-Y provided assistance for manuscript and statistical analysis.

## Conflict of Interest

CG-Y was employed by company Yidu Cloud Technology Inc. The remaining authors declare that the research was conducted in the absence of any commercial or financial relationships that could be construed as a potential conflict of interest.
